# How consistent are pre-alert guidelines? A review of UK ambulance service guidelines

**DOI:** 10.29045/14784726.2024.3.8.4.30

**Published:** 2024-03-01

**Authors:** Aimée Boyd, Fiona C. Sampson, Fiona Bell, Rob Spaight, Andy Rosser, Jo Coster, Mark Millins, Richard Pilbery

**Affiliations:** South East Coast Ambulance Service NHS Foundation Trust ORCID iD: https://orcid.org/0000-0003-1030-8167; University of Sheffield ORCID iD: https://orcid.org/0000-0003-2321-0302; Yorkshire Ambulance Service NHS Trust ORCID iD: https://orcid.org/0000-0003-4503-1903; East Midlands Ambulance Service NHS Trust ORCID iD: https://orcid.org/0000-0003-4361-5876; West Midlands Ambulance Service University NHS Foundation Trust ORCID iD: https://orcid.org/0000-0002-5477-4269; University of Sheffield ORCID iD: https://orcid.org/0000-0002-0599-4222; Yorkshire Ambulance Service NHS Trust; Yorkshire Ambulance Service NHS Trust ORCID iD: https://orcid.org/0000-0002-5797-9788

**Keywords:** guidelines, paramedic, pre-alert

## Abstract

**Aims:**

Ambulance pre-alerts are used to inform receiving emergency departments (EDs) of the arrival of critically unwell or rapidly deteriorating patients who need time-critical assessment or treatment immediately upon arrival. Inappropriate use of pre-alerts can lead to EDs diverting resources from other critically ill patients. However, there is limited guidance about how pre-alerts should be undertaken, delivered or communicated. We aimed to map existing pre-alert guidance from UK NHS ambulance services to explore consistency and accessibility of existing guidance.

**Methods:**

We contacted all UK ambulance services to request documentation containing guidance about pre-alerts. We reviewed and mapped all guidance to understand which conditions were recommended for a pre-alert and alignment with Association of Ambulance Chief Executives (AACE) and Royal College of Emergency Medicine (RCEM) pre-alert guidance. We reviewed the language and accessibility of guidance using the AGREE II tool.

**Results:**

We received responses from 15/19 UK ambulance services and 10 stated that they had specific pre-alert guidance. We identified noticeable variations in conditions declared suitable for pre-alerts in each service, with a lack of consistency within each ambulance service’s own guidance, and a lack of alignment with the AACE/RCEM pre-alert guidance. Services listed between four and 45 different conditions suitable for pre-alert. There were differences in physiological thresholds and terminology, even for conditions with established care pathways (e.g. hyperacute stroke, ST segment elevation myocardial infarction). Pre-alert criteria were typically listed in several short sections in lengthy handover procedure policy documents. Documents appraised were of poor quality with low scores below 35% for applicability and overall.

**Implications:**

There is a clear need for ambulance services to have both policies and tools that complement each other and incorporate the same list of pre-alertable conditions. Clinicians need a single, easily accessible document to refer to in a time-critical situation to reduce the risk of making an incorrect pre-alert decision.

## Introduction

Ambulance clinicians can use pre-alert calls to alert receiving emergency departments (EDs) and other hospital departments of the imminent arrival of a patient who will require immediate senior clinical review. The use of pre-alerts can help EDs to prepare for the arrival of the patient and can lead to improved time-critical treatment for certain patient groups (Ahmed et al., 2019; Hunter et al., 2019; Sheppard et al., 2015). However, overuse or inappropriate use of pre-alerts can cause incivility and tension between ambulance and ED staff, and may lead to pre-alerts not being responded to appropriately (Carberry & Harden, 2016). The Association of Ambulance Chief Executives (AACE) and the Royal College of Emergency Medicine (RCEM) collaborated to produce clinical guidelines that would help to bridge uncertainty in pre-alert practice (Association of Ambulance Chief Executives & Royal College of Emergency Medicine, 2020). As part of this process, the leaders noted a lack of consistency between existing guidance and the views of ambulance and ED professionals involved in developing the guidance.

As part of a wider study designed to understand the impact of pre-alerts on the ambulance service and ED staff and patients, we undertook an appraisal of the existing ambulance service guidance on pre-alerts to identify areas of uncertainty or conflict and explore how the guidance varied between different services and from the AACE/RCEM guidelines.

## Methods

This study is part of a wider mixed methods research study. We wrote to research leads, medical directors and heads of education in all 19 UK ambulance services (those covered by the AACE/RCEM guidelines) to ask for their latest pre-alert guidance documents. We summarised the clinical conditions recommended for each ambulance service and described the guidance in terms of areas of uncertainty, accessibility, clarity and focus.

We assessed guidance quality using the AGREE II reporting checklist (AGREE Enterprise, 2017) for clinical guidelines. The checklist uses six domains, incorporating 23 questions: (1) scope and purpose; (2) stakeholder involvement; (3) rigour of development; (4) clarity of presentation; (5) applicability; and (6) editorial independence. Two appraisers assessed all of the guidelines, in a variety of formats including policies, standard operating procedures and clinical alerts. Any element that was omitted from the document was given the minimum score of 1 in the appraisal domain. There are no appraisal tools that appraise different types of documents.

## Results

We received responses from 15/19 ambulance services; two ambulance services said they had no specific pre-alert guidance; two ambulance services reported they thought there was specific guidance in their Trust but they were unable to locate it; one used the AACE/RCEM guidance; and one service provided information pertaining to the process of pre-alerts but not pre-alertable conditions.

### Clinical conditions recommended for pre-alert

We have summarised the recommended thresholds for pre-alert for a subset of conditions, which were listed most frequently in ambulance service pre-alert guidelines, in Table 1. All services that had a documented list of pre-alert conditions were included. The table illustrates significant inconsistencies in the criteria for pre-alerts and the language and terminology used, even for time-critical conditions with known care pathways, such as ST-segment elevation myocardial infarction (STEMI) (National Institute for Health and Care Excellence [NICE], 2014) or stroke (NICE, 2022). Criteria such as ‘altered physiology’, which have objective cut-offs, have different thresholds for pre-alerting across the country. Respiratory rates with a lower threshold varied between 8 and 10, with upper thresholds ranging from ≥ 25 to > 30. No two ambulance services had the same threshold for pre-alerting Glasgow Coma Scale (GCS), with nine different ways of describing a reduced GCS score being reported. Some were very vague – using the alert, confusion, voice, pain, unresponsive (ACVPU) scale – while others used more specific scores, including a GCS motor score of < 4, or a fall of > 2 since initiating patient contact.

**Table 1.** Recommended thresholds for pre-alert.

	AACE/JRCALC	AS1	AS2	AS3	AS4	AS5	AS6	AS7	AS8	AS9
Matched with RCEM/AACE guidelines	–	3/23	4/23	6/23	10/23	7/23	19/23	10/23	10/23	4/23
RR	–			RR < 10 or > 30 for adults	Abnormal breathing rate, or irregular breathing pattern (e.g. Cheyne-Stokes breathing)	RR < 10 or > 29 (for adults)	RR ≤ 8 or ≥ 25	RR ≤ 8 or ≥ 25		NEWS > 7
Chest pains	ST elevation MI complete heart block or broad complex tachycardia with adverse features (shock, syncope, heart failure, myocardial ischaemia)		Current cardiac chest pain, with abnormal ECG (e.g. heart block, BBB)	STEMI, or cardiac chest pain where cardiac cause is suspected	STEMI, or patients with signs of cardiogenic shock	STEMI, or circulatory compromise	STEMI, or incomplete heart block	STEMI	ST elevation indicative of an MI for early thrombolysis, or haemodynamically unstable with signs and symptoms of shock	
GCS	Unconscious with a GCS motor score of less than 4		Reduced ACVPU	GCS < 8	P/U on ACVPU scale, or injured with GCS motor score < 4	GCS < 14	Unconscious with GCS motor score < 4		Trauma patients with GCS < 9 or fall of > 2 since patient contact Medical patients – unconscious	
Stroke	FAST-positive stroke within timeframe for thrombolysis	Use BE-FAST standardised framework	Any new limb weakness, speech impairment, sudden change in behaviour, FAST positive	New stroke with symptom onset of no more than 4 hours	Acute stroke (FAST-positive) being transported directly to a HASU Clinical suspicion of intracranial bleed (e.g. sub-arachnoid haemorrhage)	FAST-positive stroke	FAST-positive and within time frame for thrombolysis	FAST-positive		

There was no specific pre-alert guidance available for four ambulance services and one ambulance service had no conditions consistent with RCEM/AACE guidance, so these have been omitted from this table.

AACE: Association of Ambulance Chief Executives; ACVPU: alert, confusion, voice, pain, unresponsive; BBB: blood–brain barrier; BE-FAST: balance, eyes, face, arms, speech, time; ECG: electrocardiogram; FAST: face, arms, speech, time; GCS: Glasgow Coma Scale; HASU: hyper-acute stroke unit; JRCALC: Joint Royal Colleges Ambulance Liaison Committee; MI: myocardial infarction; NEWS: National Early Warning Score; RCEM: Royal College of Emergency Medicine; RR: respiratory rate; STEMI: ST segment elevation myocardial infarction.

Few conditions were recommended for pre-alerts by multiple services, with most conditions not listed as pre-alertable by any more than three services. Conditions that were frequently reported as pre-alertable included: airway compromise; respiratory arrest; cardiac arrest; STEMI; lowered GCS score; face, arms, speech time (FAST) test positive; uncontrolled seizure / currently fitting; and obstetric emergencies. Clinician concern was listed as criteria for pre-alert in every ambulance service. There were considerable differences in guideline specificity, with some services listing no specific conditions and others listing as many as 45 separate conditions.

While all the services did indicate that the AACE/RCEM guidelines should also be consulted, there was often an overlap in the Trust-specific guidelines. Conditions such as cardiac arrest or strokes were usually mentioned in the Trust’s own guidelines, however a considerable number of conditions were omitted.

### Accessibility of pre-alert guidance

We identified significant variation in the format and accessibility of pre-alert guidance (Table 2). Only one service had a policy specifically for pre-alerts, with other services including the pre-alerts guidance within wider policies on patient care processes and ambulance personnel responsibilities during the patient journey. Policy documents focused principally on process issues around how the pre-alert should be undertaken (e.g. pressing the correct buttons on vehicle mobile data terminal systems, at hospital and collecting hospital staff handover pins in a timely manner) to enable accurate documentation of hospital handover and ambulance turnaround times, rather than the clinical conditions that required a pre-alert. Many policies also documented in detail how handovers should be escalated should they be breaching the set 15-minute target, and some services had separate policies dedicated to this.

**Table 2.** Accessibility of pre-alert guidance.

	AS2		AS3	AS4		AS5	AS6	AS10
**Name of document**	Managing the conveyance of patients, policy and procedure	Requesting clinical support and advice	Hospital standby/pre-alert information	ED pre-arrival alert criteria for ambulance staff	ED pre-arrival alert criteria	ATMIST early and pre-alerts	Pre-alerting of patients	Clinical handover and transfer of care procedure
**Date of issue**	May 2019	August 2021	April 2015	February 2019	January 2019	February 2018	October 2020	November 2019
**Type of document**	Policy	Clinical update	Clinical update	Clinical update	Reference table	Clinical update	Clinical update	Policy
**Mnemonics used**	CASMEET	SBAR	ASHICE	Avoid SBAR, re-introducing ATMIST		ATMIST	ATMIST	ASHICE
**Who makes the pre-alert?**	Conflicting information. Clinician calls EOC – passes pre-alert onto receiving ED. Also states clinician pre-alert receiving unit directly	Clinician on scene	Inform EOC or trauma cell – does not define who will pass on the message	Ambulance staff (presumed on-scene clinician)		Lead ambulance clinician	Specifically notes EOC do NOT pass pre-alerts on from ambulance clinician. Medical – ambulance clinician; trauma – ATMIST to RTD	Most senior clinician
**When?**		When requested, or when additional clinical support required		ASAP with follow-up call 10 minutes prior to arrival		ASAP (even if not yet mobile – just state you are not yet mobile)	ASAP	
**Reference to JRCALC?**	No	Yes – use checklist (could be for cardiac arrest, or SBAR – no specific criteria listed)	No	No	No	No – states it replaces JRCALC guidelines (others are usually in conjunction with)	Yes	No

	AS8	AS9	AS11		AS12	SMTN	RCEM
**Name of document**	Hospital pre-alert and patient handover	Ambulance pre-alert process clinical number 27	Assessment, conveyance and referral of patients (emergency operations)	Pre-alert and handover guidance	Conveyance and referral policy	UK NHS ambulance services pre-alert guideline for the deteriorating adult patient	UK NHS ambulance services pre-alert guideline for the deteriorating adult patient
**Date of issue**	May 2010	July 2020	March 2017	September 2019	April 2021	September 2020	September 2020
**Type of document**	SOP	Instructions and procedures	Policy	Policy	Policy	?	Guideline
**Mnemonics used**	ATMIST SBAR ASHICE	ATMIST ASSET	MTCTC ATMIST SBAR NEWS	MTCTC SBAR ATMIST NEWS2		ATMIST SBAR	ATMIST SBAR ED
**Who makes the pre-alert?**	Crew to EMS control – EMS control to receiving hospital department	Operational/tactical commander > attending clinician > emergency vehicle operator	Clinician Must also pre-alert MTCTC for major trauma	Clinician Must also pre-alert MTCTC for major trauma	Ambulance crew	Ambulance clinician	Ambulance clinician
**When?**			10 minutes before reaching the receiving hospital	10 minutes before reaching the receiving hospital			
**Reference to JRCALC?**	Yes		No	No		JRCALC guidelines	N/A

Services that sent no guidelines have been excluded.

Clinical update * – or equivalent. A short email memo, usually sent via bulk email to clinicians.

ASAP: as soon as possible; ASHICE: age, sex, history, injuries/illness, condition, ETA; ASSET: age, signs, symptoms, ETA, treatment; ATMIST: age (name and DOB), time of onset, mechanism of injury/medical complaint, injuries, signs, treatment given; CASMEET: call-sign, age, sex, mechanism of injury/mode of illness, examination, ETA, treatments already provided; ED: emergency department; EMS: emergency medical services; EOC: emergency operations centre; JRCALC: Joint Royal Colleges Ambulance Liaison Committee; MTCTC: major trauma centre triage co-ordinator; NEWS: National Early Warning Score; RCEM: Royal College of Emergency Medicine; RTD: red trauma desk; SBAR: situation, background, assessment, recommendations.

The information specific to pre-alerts was usually a small section, buried in a lengthy policy which is inaccessible to a clinician in a time-critical scenario. Very few services replicated this information in a ‘tool’ that would be easy to use while treating a patient. The services that did have a tool did not refer to this in their policies or add the tool as an appendix.

When requesting information, the staff contacted – including research leads, medical directors and heads of education – were sometimes unable to locate the pre-alert guidance, not knowing how or where to find it or whether guidance existed. If clinicians are unable to easily locate policies they are supposed to use, they will not be able to use them and are at risk of being disciplined should they deviate from the policy. In addition, some policies defined additional, non-clinical conditions for which a pre-alert was required, for example when attending hospital with multiple patients from one incident, when there are child safeguarding concerns, when patients are attending with police or, in one service, when a patient requires a bed.

### Quality of guidance

The AGREE II checklist is intended to evaluate guidelines rather than policies or tools, but was used to identify areas where future guidance could be improved. Where trusts submitted multiple documents, we used the document that listed pre-alert conditions rather than just processes.

Assessment of quality identified variation in quality of guidance as assessed by the AGREE II tool (Table 3). However, there were clear differences in quality of guidance across wider domains. Guidance could score well across the wider domains but lack usability from an ambulance clinician perspective. For example, one 70-page policy included full details of how the guidance was developed, but lacked accessibility or clarity of criteria for an ambulance clinician to reference on scene. Appraisers were asked if they would use the tool. The tools they reported they would use had lower overall ratings.

**Table 3.** Mean appraisal scores for each domain for each document.

Domain	AS1	AS2	AS3	AS4	AS5	AS6	AS7	AS8	AS9	AS10	AS12	AS13	AACE/RCEM
1. Scope and purpose	14	57	39	8	81	83	53	72	47	39	64	0	53
2. Stakeholder involvement	0	27	8	0	11	42	50	14	8	14	41	0	33
3. Rigour of development	13	21	2	2	4	4	4	6	2	4	8	2	5
4. Clarity of presentation	11	30	52	63	56	58	58	50	39	69	36	11	78
5. Applicability	0	6	10	16	33	44	25	21	29	2	14	8	18
6. Editorial independence	0	0	0	0	0	0	0	0	0	0	0	0	0
Appraiser approved	No	No	Yes	Yes	Yes	Yes	Yes	Yes	No	Yes	No	No	Yes
All	6	23	19	15	31	38	31	27	21	21	27	4	31

Scores are out of 100. Score of < 25 is a good score.

One additional ambulance service was omitted as no guidance or policies were sent.

Where trusts submitted multiple documents, the document that detailed specific pre-alert conditions was used.

AACE: Association of Ambulance Chief Executives; RCEM: Royal College of Emergency Medicine.

Due to the nature of how policies are written, they scored better in many domains of the AGREE II tool, despite not being as user friendly. Guidance that scored better (25/100 or more) was user friendly and provided key information in an easy-to-read form at the beginning of the document. Undertaking the quality assessment identified simple ways in which the guidance could be improved. For example, the AACE/RCEM guidance does not provide detail of who needs to undertake the pre-alert, does not state who the guidance is for and loses quality marks as it cross-references other tools (JRCALC) and local policy.

## Discussion

We identified significant differences in content, quality and accessibility of guidance across UK ambulance services. Despite the importance of undertaking pre-alerts consistently and appropriately, the criteria for pre-alerts differed considerably between ambulance services, with a wide range of reference values used and a lack of consistency in acronyms. Such differences lead to postcode lotteries, with patients needing to be more unwell with worse physiological observations in some areas than in other areas in order to hit pre-alert thresholds for immediate intervention upon arrival at hospital. Moreover, the lack of consistency in terminology between ambulance services leads to breakdowns in communication when pre-alerting hospital staff, as the language used is not the same. This will be increasingly likely when receiving units on borders receive pre-alerts from multiple ambulance services.

Current pre-alert guidance in some ambulance services focuses largely on the technological processes of pre-alerting and handover (i.e. measurable process issues), rather than patient care. Although timely care is key, and government targets are important for measuring service availability, this does not measure the quality of the care provided to the patient during the interaction. The quantity of text focused on the clinical side of pre-alerts, versus the processes surrounding pre-alerts and handover, implies that meeting government targets is of higher importance than providing high-quality patient care. Going forward, we need to ensure we create policies and tools that are accessible for a patient-facing clinician, and specific to treating the patient.

There is a clear need for ambulance services to have both policies and tools that complement each other and to incorporate the same list of pre-alertable conditions. The former is required for documenting the entire process, to understand how the policy was developed and by who, to track changes over time and to document the correct procedure to staff and the public. Tools are required for quick reference in a time-critical situation.

Clinicians should only have one single document to refer to in a time-critical situation, to prevent confusion and reduce risk to clinicians making an incorrect pre-alert decision by not using the policy, tool and guidance. Individual ambulance services may add region-specific guidance, but should not omit conditions from the AACE/RCEM list of pre-alert conditions on their own tools. Similarly, any tool should be easy for a clinician to refer to in a time-critical situation, and not refer clinicians to another piece of guidance. The tool also needs to use language that is consistent with the terminology used at the receiving hospitals, to prevent confusion and ambiguity.

## Acknowledgements

We would like to thank all ambulance services for providing data. We would also like to thank Abhishek Ghosh, Imogen Roberts and Rachel Byrne for undertaking the second review of the guidance.

## Author contributions

AB undertook data collection, led the analysis and drafted the paper. FCS conceived and designed the study, contributed to analysis, drafted the paper and approved the final version. FB and MM conceived the study, helped with the study design, critically revised the paper and approved the final version. RP, AR, RS and JC contributed to acquisition, analysis and interpretation of the data, critically revised the paper and approved the final version. AB acts as the guarantor for this article.

## Conflict of interest

RP is the previous editor-in-chief of the *BPJ* (2016–2022).

## Ethics

Ethical approval for the pre-alerts project has been obtained from Newcastle & North Tyneside 2 Research Ethics Committee (Ref: 21/NE/0132).

## Funding

This project was supported by the National Institute for Health and Care Research (NIHR). The views expressed in this publication are those of the authors, and not necessarily those of the NIHR or the UK Department of Health and Social Care.
